# Bone mineral density as a prognostic factor for curve progression in adolescent idiopathic scoliosis: a longitudinal validation study

**DOI:** 10.1186/1748-7161-10-S1-O9

**Published:** 2015-01-19

**Authors:** Zhi Wei Wang, Vivian Wing Yin Hung, Huan Xiong Chen, Kwong Man Lee, Yuk Wai Lee, Bobby Kin Wah Ng, Jack Chun Yiu Cheng, Tsz Ping Lam

**Affiliations:** 1 Department of Orthopaedics and Traumatology, The Chinese University of Hong Kong, Hong Kong; 2 Lee Hysan Clinical Research Laboratories, The Chinese University of Hong Kong, Shatin, Hong Kong; 3 Joint Scoliosis Research Center of the Chinese University of Hong Kong and Nanjing University, China

## Objective

Association of adolescent idiopathic scoliosis (AIS) with low bone mineral density (BMD) has been reported with osteopenia being found to be an important prognostic factor for curve progression in AIS. The objective of this replicate study is to evaluate the predictive ability of bone mineral density on curve progression through longitudinal follow up of a new cohort.

## Methods

A cohort of 60 patients with a mean age of 12.6 years old (SD=0.8) and an average Cobb angle of 21.4 (SD=6.3) degrees were followed from their first visit till skeletal maturity defined as Risser 4 or above and greater than 1.5 years postmenarchal. Progressive curves were defined as a cumulative increase of 6 degrees or more in the Cobb angle, or the magnitude of any curve exceeding 45 degrees or the patients receiving surgery recommendation before or at skeletal maturity. BMD was measured with Dual Energy X-ray Absorptiometry of bilateral femoral necks at the first visit. Other anthropometric measurements such as body weight, body height and Tanner staging were also recorded at baseline.

## Results

The mean follow-up period was 2.6 (SD=0.7) years. The prevalence of osteopenia at the concave femoral neck was 25%. At the final follow up, 43 patients (71.7%) had curve progression, and 3 exceeded the magnitude of 45 degrees. The patients with curve progression had lower age at the first visit (p=0.03), larger initial Cobb angle (p<0.001), and lower Z-score (p=0.04) at the concave side when compared with those having stable curves. Using binary logistic regression analysis with the backward method, age at the first visit (B=-1.687, p=0.04), initial Cobb angle (B=0.434, p=0.005), and concave side Z-score (B=-2.350, p=0.008) were found to be the significant prognostic factors for curve progression.

## Conclusion

The study showed that Z-score at the concave femoral neck taken at the initial visit was distinctly lower for those with progressive curves. By following a new cohort, BMD remained an independent and significant prognostic factor for curve progression in AIS. BMD measurements should be considered during initial work up for AIS patients for purpose of prognostication. Replication of the findings that low BMD was associated with curve progression indicated its potential link with the etiopathogenesis of AIS. How this is related to deterioration in curve deformity and the underlying mechanism that lead to low bone mass in AIS warrants further studies.

## Funding source

GRF of RGC of Hong Kong (Project no: 468809).

